# Association of nocturia with cardiovascular and all-cause mortality: a prospective cohort study with up to 31 years of follow-up

**DOI:** 10.3389/fpubh.2023.1292362

**Published:** 2023-12-21

**Authors:** Min Chen, Wangan He, Shaoqian Cai, Zhi Chen, Huarong Ye, Zhigang Jin, Xuexiang Lv

**Affiliations:** ^1^Wuhan University of Science and Technology Medical College, Wuhan, China; ^2^Department of Cardiology, China Resources and Wisco General Hospital, Wuhan University of Science and Technology, Wuhan, China; ^3^China Resources and Wisco General Hospital, Wuhan University of Science and Technology, Wuhan, China

**Keywords:** nocturia, cardiovascular mortality, all-cause mortality, National Health and Nutrition Examination Survey, cohort study

## Abstract

**Background:**

Nocturia is a highly prevalent and under-considered condition and impacts the quality of life for many individuals. The long-term impact of nocturnal voiding on mortality, especially mortality from cardiovascular disease, remains unknown. The current study aimed to evaluate the relationship of nocturnal voiding episodes with cardiovascular and all-cause mortality among adults in the United States.

**Methods:**

This is a prospective cohort study of a nationally representative sample of 13,862 U.S. adults aged 20 years or older who participated in the National Health and Nutrition Examination Survey III (1988–1994). Nighttime urination frequency was reported during an in-house interview. All-cause and cause-specific mortality were ascertained by linking to National Death Index mortality data through December 31, 2019. The associations of nocturia with cardiovascular and all-cause mortality were estimated using weighted Cox proportional hazards regression models.

**Results:**

Throughout a median follow-up of 26.7 years, 5,029 deaths were reported, comprising 1,720 deaths from cardiovascular disease. In the fully adjusted model, participants who reported once, twice, and three or more times nocturnal voiding episodes have a higher risk of cardiovascular mortality (HR1, 1.22 [95% CI, 0.997–1.49], HR2, 1.47 [95% CI, 1.13–1.91], and HR ≥ 3, 1.96 [95% CI, 1.52–2.53]) as well as all-cause mortality (HR1, 1.12 [95% CI, 0.90–1.39], HR2, 1.54 [95% CI, 1.23–1.93], and HR ≥ 3, 2.48 [95% CI, 1.81–3.40]), compared to those without nocturia, and heart disease-specific mortality (HR_1_, 1.33 [95% CI, 1.08–1.64], HR_2_, 1.62 [95% CI, 1.25–2.10], and HR_≥3_, 2.07 [95% CI, 1.61–2.67]). Nevertheless, there was no significant relationship between the number of nocturia episode changes and stroke-specific mortality.

**Conclusion:**

Nocturia was associated with a significantly augmented risk of overall and heart disease-specific mortality in a dosage-dependent manner. Early recognition and taking precautions may benefit individuals with nocturia by promoting quality of life and cardiac health.

## Introduction

Frequent nighttime urination, or nocturia, is a usual complaint experienced by both men and women and has an intense influence on a patient’s health and quality of life (1, 2). In younger adults (aged 20–40 years), the prevalence of nocturia is estimated to be 11.0–35.2% in men and 20.4–43.9% in women, while these proportions are more than doubled in the older adult (3, 4). Nocturia increases the risk of falls and related injuries, resulting in annual direct economic losses of $1.5 billion in the United States (5). Meanwhile, the indirect costs, such as decreased workplace productivity and work absenteeism, are estimated to be up to $61 billion annually (5). Given its high prevalence and pernicious impact on the health and economy, nocturia is a public health issue worthy of concern.

Nocturia is associated with sleep fragmentation (6), obesity (7), hypertension (8), diabetes (9), cardiovascular morbidity (10), and all-cause mortality (11, 12). However, the effect of nocturia on mortality, especially mortality from cardiovascular disease (CVD), remains poorly understood. Most studies of nocturia and various cardiovascular risk factors have been cross-sectional or limited to populations with short follow-up periods (9–11, 13–16). Hence, identifying whether or not nocturia is an early manifestation or a complication of these conditions in the general population is essential. The long-term impact of nocturia on cardiovascular mortality based on the paucity of evidence-based data remains unclear.

The study primarily aims to determine the relationship of nocturia with cardiovascular and all-cause mortality in a nationally representative cohort with up to 31 years of follow-up in the United States.

## Methods

### Study population

The National Health and Nutrition Examination Survey (NHANES) is a large-scale, multistage, continuous, and nationwide descriptive health survey of the U.S. population led by the National Center for Health Statistics (NCHS) at the Centers for Disease Control and Prevention (CDC). NHANES III was done in two 3-year phases (1988–1991 and 1991–1994) that represented the entire civilian non-institutionalized population, age of ≥2 months, in the 50 states along with the District of Columbia of the United States. A total of 39,695 subjects were selected. Among them, interviews for 33,994 (86%) were conducted in their houses by qualified staff, 78% (30,818) were inspected in the mobile examination center, and an extra 493 individuals received a particular, partial checkup in their houses. Comprehensive explanation of NHANES III processes, interviews, and questionnaires, as well as data compilation, quality control methods, survey design, nonresponse, and sample weighting were provided (17, 18). NHANES III has acquired approval from the NCHS Ethics Review Board. Written informed consent was acquired from all participants.

We included 16,543 participants aged ≥20 years with accessible nocturia and mortality data. Participants who were pregnant (*n* = 286), died within 12 months of their NHANES III health checkup (*n* = 199), had prostate surgery (*n* = 406), or had a history of CVD or cancer (*n* = 1,790) at the baseline were excluded. Finally, 13,862 participants were included as investigative samples.

### Ascertainment of deaths

We utilized the NHANES III Public-use Linked Mortality File through 31 December 2019, which was connected by the NCHS to the National Death Index (NDI) with a corresponding algorithm to establish the mortality status (19). NDI is an NCHS-centralized databank of all demises in the United States beginning in 1979. Data on the reason for death were utilized for case description as per the 9th Revision International Statistical Classification of Diseases (ICD-9) through 1998. Moreover, the remaining information was employed for case description as per the 10th Revision (ICD-10). Ultimate causes of deaths arising before 1999 were recoded into analogous ICD-10-based core reason of death groups to adjust for the alterations between the two coding systems (19). NCHS classifies mortality from heart diseases and cerebrovascular disease (i.e., stroke) as per the ICD-10 (20). We described deaths from CVD as a result of either heart disease or cerebrovascular disease. Follow-up of all participants was done till 31 December 2019 until death except for the cases that died due to other reasons than CVD. The follow-up duration for each individual was computed as the change between the NHANES III checkup date and the final known date alive or concealed from the NHANES III mortality report.

### Exposure measurement

Each participant was questioned, “How many times a night do you usually get up to urinate (pass water)?” throughout the interview in-house, and the probable answers comprised “none, once, twice, and 3 or more times.”

### Covariate assessments

Details regarding age, sex, race/ethnicity, family income, smoking status, alcohol intake, and physical activity were acquired through standard questionnaires in the interview process. Race/ethnicity was categorized as non-Hispanic white, non-Hispanic black, Mexican American, or other. Marital status was classified as married (married and living as married), widowed, divorced, and single (never married and separated). Family income-to-poverty ratios (IPR) were classified as ≤1.30, 1.31–3.50, and > 3.50. A high IPR indicates a high family income status. Participants were labeled as non-smokers, past smokers, and current smokers according to their answers regarding smoking at least 100 cigarettes throughout their lifetime and whether or not they are presently smoking. Alcohol consumption was described depending on the answers to two survey queries on the number of drinking days over the last 12 months and the number of drinks/day on a particular drinking day (21). Current alcohol intake was classified as none (0 g/day), moderate (0.1–27.9 g/day for men and 0.1–13.9 g/d for women), as well as heavy drinking (≥ 28 g/day for men and ≥ 14 g/d for women) (22). For physical activity, the inactive group was described as those with no documented leisure time physical activity, and the active group was defined as those who had suggested levels of physical activity {i.e., self-reported leisure-time moderate activity [metabolic equivalents (METs) with a range from 3 to 6] of five or more times per week or leisure time vigorous activity (METs >6) three or more times per week}. The insufficiently active group was described as the ones who were not inactive and did not fulfill the criteria for suggested levels of physical activity (23). Total energy intake (TEI) was computed by the United States Department of Agriculture Automated Multiple-Pass Method. We utilized the Healthy Eating Index (HEI)-2010 to reveal the overall quality of diet (HEI-2010 score from 0 to 100, where 100 indicates the best-quality diet) (24). Obesity, hypertension, diabetes, and dyslipidemia are the main risk factors for CVD. Measurements of height and weight were done as per a standard protocol. Body mass index (BMI) was computed as weight (kg) divided by height (meters squared). Hypertension was described as taking antihypertensive medicines or having a systolic blood pressure (SBP) level of ≥130 mmHg or a diastolic blood pressure (DBP) level of ≥80 mmHg as per the 2017 ACC/AHA Hypertension Guideline (25). Diabetes was described as any participant recognized with diabetes, taking insulin, taking diabetes pills, having a hemoglobin A1c level of ≥6.5%, or having a fasting plasma glucose level of ≥126 mg/dL (26). Dyslipidemia is characterized by an abnormal lipid profile that includes elevated levels of total cholesterol, low-density lipoprotein (LDL) cholesterol, triglyceride (TG), and lowered high-density lipoprotein (HDL) cholesterol concentrations. In our study, patients with dyslipidemia were characterized as a self-reported physician’s diagnosis, taking cholesterol-lowering medicines, or had a TG level of ≥150 mg/dL and HDL cholesterol level of <40 mg/dL based on guidelines from the third report of the National Cholesterol Education Program Adult Treatment Panel III (27).

### Statistical analysis

All data analyses responsible for the composite, multistage, stratified, and cluster-sampling design (comprising oversampling of specific subpopulations) of NHANES by utilizing sample weights, strata, and principal sampling units were entrenched in the NHANES data. Logistic regression for categorical variables and linear regression for continuous variables were utilized to compare means and baseline characteristics. The relations among nocturia episodes and cardiovascular plus all-cause mortalities were examined by utilizing Cox proportional hazard regression models with the succeeding covariates: age, sex, and race/ethnicity (Model 1); Model 1 plus marital status, family income level, smoking status, alcohol intake, physical activity, TEI, and HEI-2010 (Model 2); Model 2 plus BMI, hypertension (yes/no), diabetes (yes/no), and dyslipidemia (yes/no) (Model 3); Model 3 plus serum C-reactive protein (CRP) (Model 4). Dummy variables were used to indicate missing covariate values. The model assumptions for all the analyses were checked, and no violation was found.

Subgroup analyses were done for cardiovascular and all-cause mortality results by the following variables: sex (men, women), age (20–40, 40–60, and > 60 years), race/ethnicity (non-Hispanic white, non-Hispanic black, Hispanic, and others), the ratio of family income to poverty (≤1.30, 1.31–3.50, and > 3.50), smoking status (never, former, current), alcohol intake (non-drinker, current drinker), physical activity (inactive or insufficient, recommended level), weight status (BMI <25.0, 25.0–29.9, and ≥ 30.0 kg/m^2^), diabetes (yes/no), hypertension (yes/no), dyslipidemia (yes/no), and serum CRP level (≤1 and > 1 mg/dL). A joint test was performed to acquire a *p-*value for interaction for investigating the statistical significance of the difference among subgroups. In addition, we considered competing risks using Fine & Gray models to examine the robustness of the findings. SAS version 9.4 (SAS Institute, United States) was utilized for analyses with a two-sided significance threshold of *p* < 0.05.

## Results

### Population characteristics

Among the 13,862 participants (mean age 45.0 years, SE ± 0.2; 46.6% male) in this study, 43.2% (*n* = 5,987) never experienced nocturia, 34.0% (*n* = 4,711) reported one void per night, 14.2% (*n* = 1,972) reported two voids per night, and 8.6% (*n* = 1,198) experienced nocturia occurring three or more times per night. During the 323,968 person-years of follow-up (median follow-up 26.7 years; maximum follow-up 31 years), there were 5,029 deaths, comprising 1,720 deaths from CVD. As mentioned in Table 1, the participants who reported three or more voiding per night were likely old, unmarried, non-Hispanic black, former smokers, non-drinkers, physically inactive, and had lower family income and lesser energy intake, compared with those who had fewer nocturia episodes. As shown in Table 2, the subjects who reported three or more times nocturnal voiding were more likely to experience obesity, hypertension, and diabetes as well as had greater levels of total blood cholesterol, triglycerides, and serum CRP compared with those who reported fewer nocturia episodes.

**Table 1:** Baseline characteristics of the study population.

Characteristics	No. nocturia episodes				*P*-value
	None	Once	Twice	Three or more times	
No. of participants	5,987	4,711	1,972	1,192	
Age, mean (SE), years	38.2 (0.3)	44.7 (0.6)	50.3(0.6)	54.1 (0.8)	< 0.001
Sex, % (SE)					< 0.001
Male	52.1 (0.7)	47.8 (0.8)	39.0 (1.4)	37.6 (1.8)	
Female	47.9 (0.7)	52.2 (0.8)	61.0 (1.4)	62.4 (1.8)	
Race/ethnicity, % (SE)					< 0.001
Non-Hispanic white	78.4 (1.4)	75.1 (1.2)	64.6 (2.4)	60.4 (2.7)	
Non-Hispanic black	8.1 (0.5)	12.2 (0.8)	18.4 (1.4)	24.1 (2.0)	
Hispanic	5.5 (0.4)	5.3 (0.5)	4.8 (0.5)	5.9 (0.7)	
Other	8.0 (1.0)	7.3 (0.8)	12.2 (2.1)	9.6 (1.9)	
Marital status, % (SE)					< 0.001
Married	65.7 (1.4)	66.8 (1.1)	64.1 (1.9)	54.8 (2.2)	
Widowed	3.5 (0.3)	6.1 (0.5)	9.6 (0.7)	16.8 (1.5)	
Divorced	7.4 (0.5)	9.4 (0.7)	8.5 (1.1)	10.7 (1.2)	
Single	23.3 (1.3)	17.6 (1.0)	17.8 (1.6)	17.6 (2.4)	
Missing	0.1 (0.0)	0.1 (0.1)	0.1 (0.0)	0.2 (0.1)	
Ratio of family income to poverty, % (SE)					< 0.001
≤ 1.30	13.6 (0.9)	16.6 (1.2)	25.1 (2.0)	33.0 (2.2)	
1.31-3.50	43.7 (1.4)	41.5 (1.3)	41.8 (2.0)	39.0 (2.3)	
> 3.50	37.0 (1.4)	35.6 (1.7)	24.4 (2.1)	18.0 (2.1)	
Missing	5.8 (0.5)	6.2 (0.5)	8.8 (1.5)	10.1 (1.4)	
Smoking status, % (SE)					< 0.001
Non-smoker	47.3 (1.3)	45.4 (1.2)	47.4 (1.8)	48.8 (2.3)	
Former smoker	19.9 (0.7)	27.2 (1.2)	28.3 (1.5)	25.4 (1.8)	
Current smoking	32.8 (1.1)	27.3 (1.0)	24.2 (1.8)	25.7 (1.9)	
Alcohol intake, % (SE)*					< 0.001
Non-drinker	70.1 (1.3)	71.7 (1.6)	78.7 (1.8)	81.2 (2.0)	
Moderate drinking	9.4 (0.6)	9.2 (0.9)	5.9 (0.7)	6.1 (1.6)	
Heavy drinking	17.7 (0.8)	15.7 (1.0)	10.5 (1.3)	9.5 (1.2)	
Missing	2.8 (0.6)	3.5 (0.6)	4.9 (1.2)	3.2 (0.9)	
Physical activity, % (SE)†					< 0.001
Inactive	11.3 (0.8)	13.5 (0.8)	22.1 (1.4)	26.1 (2.5)	
Insufficient	47.4 (1.1)	44.3 (1.6)	43.1 (1.8)	38.4 (2.6)	
Recommended level	41.3 (1.2)	42.3 (1.5)	34.8(1.9)	35.5 (2.4)	
TEI, mean (SE), kcal/day	2278.0 (24.4)	2105.4 (32.6)	1890.7 (35.8)	1821.1 (44.9)	< 0.001
HEI-2010, mean (SE)	62.9 (0.3)	63.7 (0.4)	63.8 (0.6)	63.5 (0.8)	< 0.001

Values are means (SE) or percentages (SE) and are weighted except no. of participants. HEI, healthy eating index; SE, standard errors; TEI, total energy intake. *Non-drinker: 0 g/day; Moderate drinking: 0.1–28 g/day for men and 0.1–14 g/d for women; Heavy drinking: ≥ 28 g/day for men and ≥ 14 g/d for women. †Insufficient activity was defined where the sum of (weekly frequency of moderate activity/5) + (weekly frequency of vigorous activity/3) is <1, and recommended activity was defined where the sum of (weekly frequency of moderate activity/5) + (weekly frequency of vigorous activity/3) is ≥1.

**Table 2:** Distribution of CVD risk factors of the study population.

CVD risk factors	No. nocturia episodes				*P*-value
	None	Once	Twice	Three or more times	
No. of participants	5,987	4,711	1,972	1,192	
BMI categories, % (SE)					< 0.001
BMI (kg/m^2^) < 25.0	50.8 (1.1)	43.4 (1.2)	33.6 (1.7)	31.7 (2.1)	
25.0 ≤ BMI (kg/m^2^) <29.9	31.3 (0.9)	32.0 (1.0)	35.5 (1.8)	30.8 (1.7)	
BMI (kg/m^2^) ≥ 30.0	17.9 (0.8)	24.6 (1.1)	30.7 (1.6)	37.5 (2.1)	
Missing	0.1 (0.0)	0.1 (0.0)	0.2 (0.1)	0.1 (0.1)	
Hypertension, % (SE)	13.7 (0.7)	23.2 (1.2)	32.9 (1.5)	43.6 (2.4)	< 0.001
DBP, mean (SE), mm Hg	73.7 (0.2)	74.9 (0.3)	75.0 (0.3)	76.4 (0.4)	< 0.001
SBP, mean (SE), mm Hg	118.1 (0.3)	122.6 (0.6)	127.2 (0.7)	130.8 (1.1)	< 0.001
Diabetes, % (SE)	8.3 (0.4)	14.2 (1.1)	22.6 (1.6)	28.6 (2.1)	< 0.001
Fasting glucose, mean (SE), mg/dL	94.5 (0.5)	98.8 (0.7)	102.9 (1.1)	111.6 (2.4)	< 0.001
Glycated hemoglobin, mean (SE), %	5.2 (0.0)	5.4 (0.0)	5.6 (0.0)	5.9 (0.1)	< 0.001
Dyslipidemia, % (SE)	35.5 (1.3)	40.1 (1.3)	42.2 (2.0)	46.5 (2.1)	< 0.001
TC, mean (SE), mg/dL	198.2 (1.0)	204.6 (1.2)	213.1 (1.4)	209.7 (1.9)	< 0.001
LDL-C, mean (SE), mg/dL	124.6 (1.0)	128.2 (1.4)	131.3 (2.1)	129.1 (2.6)	< 0.001
HDL-C, mean (SE), mg/dL	50.4 (0.4)	50.8 (0.5)	51.2 (0.7)	50.7 (0.7)	< 0.001
TG, mean (SE), mg/dL	131.8 (2.5)	142.5 (2.1)	148.3 (4.7)	161.6 (6.8)	< 0.001
CRP, mean (SE), mg/dL	0.3 (0.0)	0.4 (0.0)	0.5 (0.0)	0.6 (0.0)	< 0.001

Values are means (SE) or percentages (SE) and are weighted except no. of participants. BMI, body mass index; CRP, serum C-reactive protein; CVD, cardiovascular disease; DBP, diastolic blood pressure; HDL-C, high density lipoprotein cholesterol; LDL-C, low density lipoprotein cholesterol; SE, standard errors; TG, triglycerides.

### Cardiovascular and all-cause mortality

**Figure 1:**
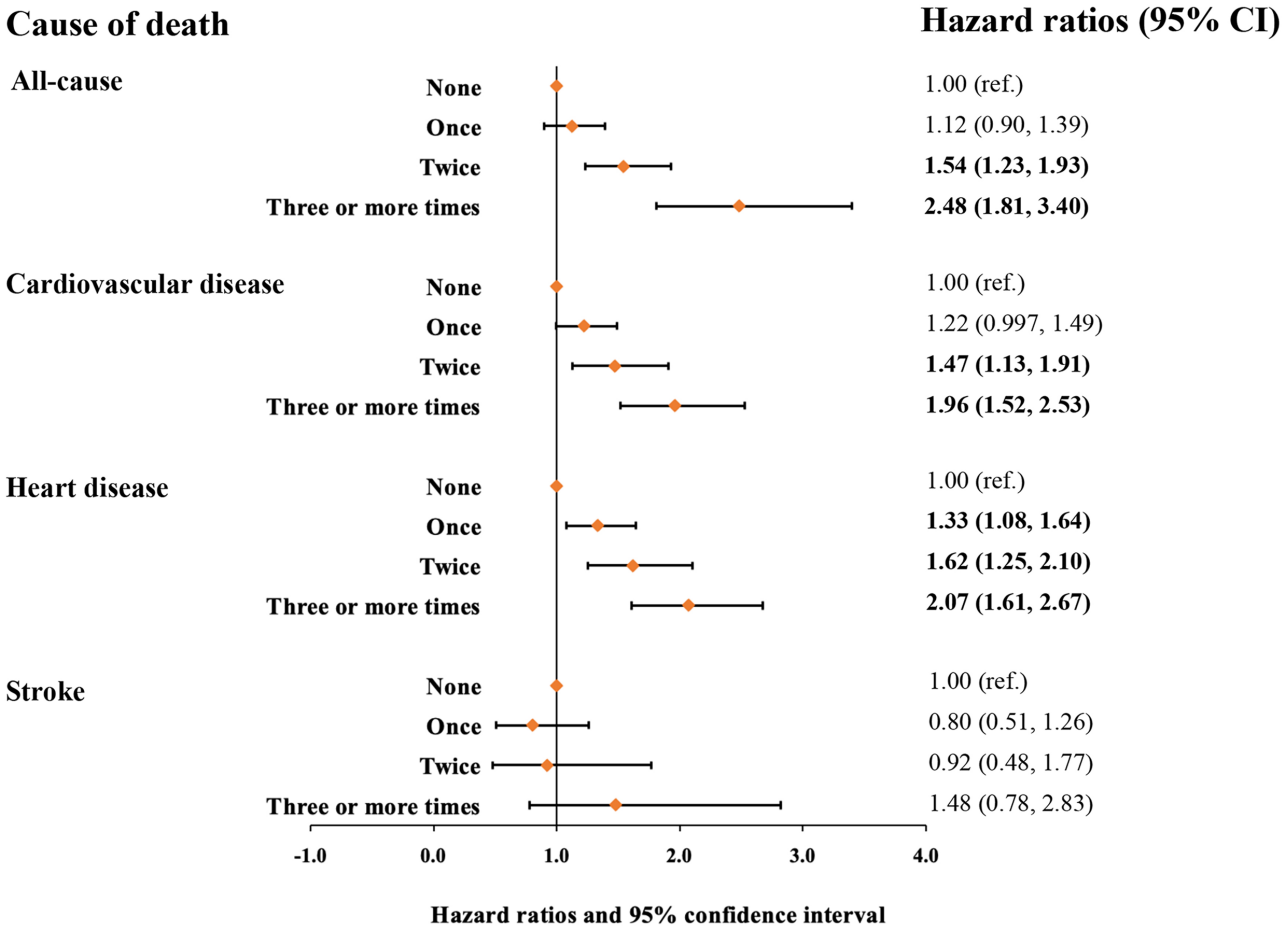
Hazard ratios for mortality from all causes, cardiovascular disease, heart disease, and stroke based on nocturia episodes. Hazard ratios were adjusted for age, sex, race/ethnicity, marital status, family income level, smoking status, alcohol intake, physical activity, TEI, overall diet quality indicated by HEI-2010, BMI, hypertension (yes/no), diabetes mellitus (yes/no), dyslipidemia (yes/no), and CRP.

Mortality risk was consistent with the increasing number of voiding episodes in a dose-dependent manner. Participants who reported three or more times voiding per night had the highest risk for all-cause and cardiovascular mortality. Following the adjustment for age, sex, and race/ethnicity (model 1), the participants with three or more times voiding per night had a 3.49-fold greater risk of all-cause mortality [hazard ratio (HR), 3.49 and 95% confidence interval (CI), 2.65–4.60] and 2.37-fold greater risk of cardiovascular mortality (HR, 2.37 [95% CI, 1.85–3.02]) compared with those without nocturia (Table 3). In the fully adjusted model (model 4), participants who reported once, twice, and three or more times nocturnal voiding episodes have a higher risk of cardiovascular mortality (HR_1_, 1.22 [95% CI, 0.997–1.49], HR_2_, HR_2_, 1.47 [95% CI, 1.13–1.91], and HR_≥3_, 1.96 [95% CI, 1.52–2.53]) as well as all-cause mortality (HR_1_, 1.12 [95% CI, 0.90–1.39], HR_2_, 1.54 [95% CI, 1.23–1.93], and HR_≥3_, 2.48 [95% CI, 1.81–3.40]), compared with those without nocturia (Table 3 and Figure 1).

**Table 3:** Association of nocturia with cardiovascular and all-cause mortality.

	No. nocturia episodes			
	None	Once	Twice	Three or more times
Cardiovascular disease mortality				
Deaths/person years	459/151,288	615/109,986	345/40,372	301/22,320
Unadjusted	1.00	2.16 (1.78–2.61)	3.61 (2.89–4.52)	6.55 (5.24–8.20)
Model 1	1.00	1.24 (1.01–1.52)	1.57 (1.22–2.03)	2.37 (1.85–3.02)
Model 2	1.00	1.30 (1.05–1.60)	1.59 (1.24–2.04)	2.21 (1.72–2.85)
Model 3	1.00	1.23 (1.00–1.51)	1.49 (1.15–1.92)	2.00 (1.55–2.57)
Model 4	1.00	1.22 (0.997–1.49)	1.47 (1.13–1.91)	1.96 (1.52–2.53)
All–cause mortality				
Deaths/person years	1429/151288	1816/109986	1048/40372	736/22320
Unadjusted	1.00	1.93 (1.66–2.25)	3.13 (2.70–3.62)	4.77 (4.11–5.53)
Model 1	1.00	1.14 (0.93–1.41)	1.73 (1.38–2.18)	3.49 (2.65–4.60)
Model 2	1.00	1.14 (0.93–1.41)	1.64 (1.28–2.09)	2.96 (2.1–4.18)
Model 3	1.00	1.13 (0.92–1.39)	1.56 (1.25–1.95)	2.56 (1.86–3.53)
Model 4	1.00	1.12 (0.90–1.39)	1.54 (1.23–1.93)	2.48 (1.81–3.40)

Values are *n* or hazard ratio (95% confidence interval) and are weighted except no. of deaths/person-years. Model 1: adjusted for age, sex, and race/ethnicity. Model 2: model 1 plus marital status, family income level, smoking status, alcohol intake, physical activity, TEI, and overall diet quality indicated by HEI-2010. Model 3: model 2 plus BMI, hypertension (yes/no), diabetes mellitus (yes/no), and dyslipidemia (yes/no). Model 4: model 3 plus CRP.

### CHD and stroke mortality

Next, we assessed the relationship of nocturia with mortality from heart disease and stroke (Table 4 and Figure 1). Compared with those without nocturia, the participants who had three or more times voiding episodes per night had a greater risk of heart disease-specific mortality (HR, 2.56 [95% CI, 1.97–3.34]) in models adjusted for age, sex, race/ethnicity (model 1). In the fully-adjusted model (model 4), the connection between nocturia and heart disease-specific mortality was somewhat diminished but remained substantial (HR_1_, 1.33 [95% CI, 1.08–1.64], HR_2_, 1.62 [95% CI, 1.25–2.10], and HR_≥3_, 2.07 [95% CI, 1.61–2.67] for once, twice, and three or more times vs. none). Nevertheless, the relationship between nocturia and stroke-specific mortality was not substantial.

**Table 4:** Association of nocturia with heart disease and stroke mortality.

	No. nocturia episodes			
	None	Once	Twice	Three or more times
Heart disease mortality				
Deaths/person years	366/151,288	505/109,986	278/40,372	246/22,320
Unadjusted	1.00	2.31 (1.89–2.83)	3.88 (3.06–4.92)	6.78 (5.40–8.52)
Model 1	1.00	1.35 (1.09–1.67)	1.73 (1.33–2.26)	2.56 (1.97–3.34)
Model 2	1.00	1.42 (1.14–1.76)	1.75 (1.35–2.25)	2.35 (1.80–3.07)
Model 3	1.00	1.34 (1.08–1.65)	1.63 (1.26–2.11)	2.11 (1.63–2.72)
Model 4	1.00	1.33 (1.08–1.64)	1.62 (1.25–2.10)	2.07 (1.61–2.67)
Stroke mortality				
Deaths/person years	93/151288	110/109986	67/40372	55/22320
Unadjusted	1.00	1.55 (1.01–2.38)	2.58 (1.50–4.45)	5.64 (3.31–9.63)
Model 1	1.00	0.82 (0.53–1.27)	0.99 (0.55–1.81)	1.66 (0.95–2.90)
Model 2	1.00	0.84 (0.54–1.31)	0.98 (0.53–1.82)	1.64 (0.88–3.07)
Model 3	1.00	0.82 (0.52–1.29)	0.95 (0.50–1.81)	1.55 (0.82–2.91)
Model 4	1.00	0.80 (0.51–1.26)	0.92 (0.48–1.77)	1.48 (0.78–2.82)

Values are *n* or hazard ratio (95% confidence interval) and are weighted except no. of deaths/person-years. Model 1: adjusted for age, sex, and race/ethnicity. Model 2: model 1 plus marital status, family income level, smoking status, alcohol intake, physical activity, TEI, and overall diet quality indicated by HEI-2010. Model 3: model 2 plus BMI, hypertension (yes/no), diabetes mellitus (yes/no), and dyslipidemia (yes/no). Model 4: model 3 plus CRP.

### Sensitivity analyses

We performed a subgroup analysis to find the identification of potential special populations and the optimal range of interaction effects. The association between nocturia and all-cause mortality was greater in male participants (adjusted HR_≥3_, 2.15 [95% CI, 1.59–2.90]) than in female participants (adjusted HR_≥3_, 1.50 [95% CI, 1.20–1.88]) (*p*-value for interaction, 0.013) and participants of 20 to 40 years old (adjusted HR ≥ _3_, 2.45 [95% CI, 1.53–3.93]) than participants over 60 years old (adjusted HR_≥3_, 1.44 [95% CI, 1.18–1.76]) (*p*-value for interaction, <0.0001) (Supplementary Table S1; Table 5), more substantial in Non-Hispanic white (adjusted HR_≥3_, 1.97 [95% CI, 1.53–2.55]) as well as Hispanic (adjusted HR_≥3_, 1.75 [95% CI, 1.33–2.29]) than other races (*p*-value for interaction, 0.023), and more robust in non-diabetes (adjusted HR_≥3_, 1.79 [95% CI, 1.42–2.25]) than individuals with diabetes (adjusted HR_≥3_, 1.70 [95% CI, 1.24–2.33]) (*p*-value for interaction, 0.11). The relationship between nocturia and cardiovascular mortality seemed greater in participants with BMI lesser than 25 kg/m^2^ (adjusted HR_≥3_, 2.44 [95% CI, 1.67–3.57]) compared to overweight (BMI ≥25 to <30 kg/m^2^; adjusted HR_≥3_, 1.73 [95% CI, 1.09–2.76]) or obese participants (BMI ≥30 kg/m^2^; adjusted HR_≥3_, 1.74 [95% CI, 1.13–2.69])(*p*-value for interaction, 0.025). In addition, the relationship between nocturia and cardiovascular mortality seemed greater in male participants than in female participants and participants of 20–40 years old than participants over 60 years old (Supplementary Table S2; Table 5).

**Table 5:** Association between nocturia and mortality stratified by age-sex specific stratifications.

	No. nocturia episodes			
	None	Once	Twice	Three or more times
All-cause mortality				
Age < 60 and men	1.00	1.82 (1.42, 2.34)	2.54 (1.91, 3.38)	3.56 (2.46, 5.14)
Age < 60 and women	1.00	1.19 (0.91, 1.57)	1.27 (0.97, 1.65)	1.67 (1.16, 2.40)
Age ≥ 60 and men	1.00	1.09 (0.90, 1.31)	1.25 (0.96, 1.64)	1.40 (1.03, 1.91)
Age ≥ 60 and women	1.00	1.04 (0.88, 1.22)	1.48 (1.23, 1.79)	1.51 (1.20, 1.91)
Cardiovascular disease mortality				
Age < 60 and men	1.00	1.62 (1.13, 2.31)	2.09 (1.10, 3.97)	2.75 (1.53, 4.94)
Age < 60 and women	1.00	1.80 (1.02, 3.17)	1.65 (0.83, 3.27)	2.84 (1.46, 5.50)
Age ≥ 60 and men	1.00	1.20 (0.85, 1.70)	1.64 (1.15, 2.35)	2.13 (1.30, 3.47)
Age ≥ 60 and women	1.00	0.94 (0.74, 1.19)	1.13 (0.78, 1.63)	1.51 (1.10, 2.09)
Heart disease mortality				
Age < 60 and men	1.00	1.67 (1.15, 2.43)	2.02 (0.997, 4.07)	2.59 (1.47, 4.56)
Age < 60 and women	1.00	2.10 (1.20, 3.67)	1.77 (0.96, 3.28)	3.18 (1.59, 6.36)
Age ≥ 60 and men	1.00	1.11 (0.76, 1.63)	1.67 (1.15, 2.43)	2.11 (1.26, 3.55)
Age ≥ 60 and women	1.00	1.08 (0.81, 1.43)	1.32 (0.92, 1.90)	1.63 (1.14, 2.34)
Stroke mortality				
Age < 60 and men	1.00	1.37 (0.51, 3.72)	2.93 (0.70, 12.38)	4.62 (0.88, 24.22)
Age < 60 and women	1.00	0.85 (0.22, 3.24)	1.06 (0.16, 7.05)	2.95 (0.85, 10.23)
Age ≥ 60 and men	1.00	2.25 (0.89, 5.74)	1.25 (0.40, 3.95)	2.47 (0.65, 9.39)
Age ≥ 60 and women	1.00	0.59 (0.34, 1.03)	0.67 (0.31, 1.42)	1.22 (0.55, 2.70)

Values are n or hazard ratio (95% confidence interval) and are weighted except NO. of deaths/person-years. Data were presented as hazard ratios (95% CIs) with adjustment of age, sex, race/ethnicity, marital status, family income level, smoking status, alcohol intake, physical activity, TEI, and overall diet quality indicated by HEI-2010, BMI, hypertension (yes/no), diabetes mellitus (yes/no), and dyslipidemia (yes/no), CRP.

We conducted sensitivity analyses after excluding participants who died within 2 years of follow-up, missing covariates and BMI ≥ 40.0 kg/m^2^ (Supplementary Table S3) and found that the results remained robust. Meanwhile, the results of competing risk analysis for nocturia and cause-specific mortality are shown in Supplementary Table S4. No substantial changes were observed.

## Discussion

In the current prospective study, including 13,862 adults with a median follow-up of 26.7 years (maximum 31 years), frequent nocturnal voiding was associated with a remarkably augmented risk of cardiovascular as well as all-cause mortality and showed a graded association with the times of nocturia.

The results were consistent with the latest meta-analysis stating that the risk of all-cause mortality increases by 23% in subjects with nocturia, even though significant heterogeneity was detected across seven studies, presumably due to heterogeneous age, race, and exposure definitions (28). Most reports, including two recent studies (12, 29), on nocturia and all-cause mortality, were restricted to older adults. A previous study explored the relationship between nocturia and all-cause mortality utilizing data from NHANES III (11). However, their mortality data ended on December 31, 2000, which means that their follow-up time was much shorter than this study. Prior evidence and our findings suggested that the early appearance of nocturia may reflect a marker or clinical indicator of overall health and related mortality.

A previous study reported nocturia is a significant independent indicator of mortality among 119 70-year-old patients with ischemic heart disease (30). Our findings are consistent with those of previous studies on the relationship between nocturia and CHD occurrence. A cohort with 2,247 male U.S. residents revealed that younger men (40–60 years old) with moderate nocturia (urinate ≥ two times per night) had a 68% (unadjusted HR, 1.68 [95% CI, 1.13–2.49]) higher risk of CHD compared with men without nocturia (31). This association was no longer substantial in the adjusted model (adjusted HR, 1.36 [95% CI, 0.87–2.12]), which was probably due to the limited sample size. A population study in Finland found that the prevalence of nocturia increased at a constant rate with age. However, Nocturia increased twice as rapidly in men as among women (OR increased 7.3% [95% CI 6.5–8.2%] and 3.5% [95% CI 2.9–4.1%] per year among men and women, respectively), and this is consistent with the results of subgroup analysis in this study (32). In the Boston Area Community Health Survey, overall cardiac disease (including congestive heart failure, coronary disease, myocardial infarction, or angina) is an independent correlate for nocturia (9, 16). Another cross-sectional analysis indicated that nocturia is related to augmented odds of cardiovascular morbidity (mainly hypertension and stroke) independent of usual cardiovascular risk factors (10). Additionally, nocturia is also related to general obesity (33), increased serum CRP (34), and multiple cardiometabolic risk factors (35, 36). However, after the adjustment for these confounders, the dose-dependent association of nocturia with CHD mortality remained significant in our results, but that with stroke-specific mortality was null. In summary, previous and present findings support the prominence of nocturia as a risk factor for cardiovascular morbidity and mortality.

The mechanisms linking increased nocturia and cardiovascular mortality are uncertain, but several known causes of nocturia and related comorbidities are possible risk factors associated with poor cardiovascular outcomes. First, nocturnal voiding can negatively influence the occurrence of N3 (deep) sleep and impose circadian dysfunction (37–39). Sleep fragmentation promotes the development of atherosclerotic lesions through the neuro-immune axis and may further cause cardiovascular events and death (40). In turn, sleep-associated disorders (e.g., obstructive sleep apnea, restless leg syndrome, or periodic limb movements) are potential causes of nocturia (41, 42). Second, nocturia occurs in patients with congestive heart failure or another peripheral edema (e.g., chronic kidney disease or liver disease), which would elevate atrial natriuretic peptide (43). Nighttime recumbent position decreases crura hydrostatic pressure, increases circulatory volume and hemodilution, and suppresses antidiuretic hormone secretion, which in turn increases nocturnal voiding (44). Third, poor control of diabetes is often accompanied by polyuria and leads to nocturia. Meanwhile, uncontrolled diabetes is a significant risk factor for CVD morbidity and mortality. Fourth, nighttime overproduction of urine is caused by the impaired circadian rhythm of secretion of arginine vasopressin (AVP) or the dysregulation of AVP receptors (45). The long-term dysregulation of the AVP system has a deleterious impact on vasoconstriction and blood pressure control, which in turn promote the development and progression of CVD (46). Nocturia and CVD may partly share common pathophysiological pathways. Our findings suggested that subjects with nocturia at the baseline may have disordered cardiovascular system homeostasis. Although we excluded subjects with CVD at baseline and adjusted for CVD risk factors (including obesity, hypertension, hyperglycemia, and dyslipidemia) in the current study, the relationship among nocturia and CVD mortality, especially CHD mortality, persisted in being substantial and in a dose-dependent manner. The relationship between nocturia and adverse cardiovascular outcomes is complex and needs further research.

Nocturia is highly multifactorial in nature. Therefore, classifying nocturia as a specific symptom and recognizing it as a unique entity is important. The increasing number of consensus statements and expert reviews indicate that nocturia is a common symptom that severely impacts the quality of life for many people but has been underreported, undertreated, and misunderstood (1, 47–49). Our findings underscore the need to understand nocturia in daily life, and its early appearance may be a valuable indicator of cardiovascular and all-cause mortality. Opposite to the perception that nocturia is only a trivial condition or a “normal” consequence of the aging procedure (50), this condition must be encountered proactively and considered an avoidable syndrome. For individuals with nocturia, the potential etiology and beginning with behavioral intervention must be identified (51). Medications can be used for patients with severe conditions when necessary, especially those with other comorbidities (e.g., overactive bladder, benign prostatic hyperplasia, or obstructive sleep apnea). Optimizing lifestyle intervention, medication regimens, and careful attention to comorbid conditions may help individuals with nocturia promote quality of life and cardiovascular health.

The main strength of this study are its prospective longitudinal nature and long-term follow-up duration of up to 31 years, which might have offered more power to identify relations. The large nationally representative population helps to extend the results to the common population in the United States. Moreover, through the comprehensive data gathered in NHANES III, we are capable of regulating the possible perplexing consequences of various demographic, socioeconomic, lifestyle, and diseases, as well as performing sensitivity analyses to estimate the coherence of the findings according to participant characteristics.

Several limitations are also noteworthy. First, the effects of changes in nocturia during the follow-up on cardiovascular and all-cause mortality were unknown since the information on nocturnal voiding frequency in NHANES III was acquired only at the baseline, and participants might develop nocturia during the follow-up. In addition, the exposure assessments were derived from self-reported questionnaires, which lacked detailed information on nocturia patterns such as transient or persistent, nocturnal urine volume, and daytime voiding. Therefore, a misclassification bias may also have potentially resulted in over or underestimating mortality rates. Secondly, the NHANES III-related mortality document determines the cause of death via the connection to the National Death Index. Even though this method has previously been confirmed by the CDC and utilized in numerous CDC reports or related published evidence, we cannot exclude the probability of misclassification of the reason for death. Third, although we considered several possible confounders and exclusion criteria, it cannot completely address the residual confounding (e.g., benign prostate hyperplasia, overactive bladder, prostatitis, and urinary tract infection) and bias because of the nature of the observational study. Additionally, we did not have information on sleep duration or sleep disorders in NHANES III participants to determine the sleep interruption or quality in this population. Beyond the negative cardiovascular impact of nocturia, the interrelationship between nocturia and sleep quality and their co-effect in the progression of CVD and related mortality is also interesting.

## Conclusion

In summary, in the current prospective study, including 13,862 adults with up to a maximum follow-up of 31 years, a dose-dependent pattern of increasing mortality risk, especially from heart disease, with an increasing number of nocturnal voiding episodes was observed. Nocturia might be a potential early indicator of cardiovascular and all-cause mortality in both men and women that requires greater public health attention. Early intervention for nocturia may help prevent CVD risk. To this end, further studies in other countries or ethnic populations are warranted to evaluate whether lifestyle modification or medical treatment for nocturia will promote cardiovascular health, improve the overall quality of life, and curb the subsequent risk of death.

## Data availability statement

The datasets presented in this study can be found in online repositories. The names of the repository/repositories and accession number(s) can be found in the article/Supplementary material.

## Author contributions

MC: Formal analysis, Investigation, Methodology, Software, Writing – original draft. WH: Writing – review & editing. SC: Resources, Supervision, Writing – review & editing. ZC: Formal analysis, Writing – review & editing. HY: Writing – review & editing. ZJ: Funding acquisition, Supervision, Writing – review & editing. XL: Writing – review & editing.
