# Supplementary material for: Association of nocturia with cardiovascular and all-cause mortality: a prospective cohort study with up to 31 years of follow-up
Source: Front Public Health. 2023 Dec 21;11:1292362. doi: 10.3389/fpubh.2023.1292362 (PMC10768185; doi:10.3389/fpubh.2023.1292362)
Supplement: Supplementary file 1 [file Data_Sheet_1.pdf]

Supplemental Material

Association of Nocturia with Cardiovascular and All-Cause Mortality

—— A Prospective Cohort Study with Up to 31 Years of Follow-Up

Contents:

Supplemental Table 1. Association Between Nocturia and All-Cause Mortality among Different Subgroups

Supplemental Table 2. Association Between Nocturia and Cardiovascular Mortality among Different Subgroups

Supplementary Table 3. Sensitivity analyses of the association between Nocturia and mortality excluding those who developed died within two years of follow-up, missing covariates and BMI ≥ 40.0 kg/m^2.(n=10371)

Supplementary Table 4. Association between Nocturia and cause-specific mortality in competing-risk models

Supplemental Table 1. Association Between Nocturia and All-Cause Mortality among Different Subgroups

	No. Nocturia Episodes				***P* interaction**
	**None**	**Once**	**Twice**	**Three or more times**	
**Sex**					
Men	1.00	**1.42 (1.20-1.69)**	**1.77 (1.48-2.12)**	**2.15 (1.59-2.90)**	0.0013
Women	1.00	1.06 (0.89-1.25)	**1.34 (1.13-1.60)**	**1.50 (1.20-1.88)**	
**Age (years)**					
20-40	1.00	1.31 (0.94-1.83)	1.23 (0.88-1.72)	**2.45 (1.53-3.93)**	<0.0001
40-60	1.00	**1.45 (1.12-1.88)**	**1.88 (1.45-2.42)**	**2.20 (1.56-3.11)**	
> 60	1.00	1.04 (0.92-1.19)	**1.37 (1.16-1.62)**	**1.44 (1.18-1.76)**	
**Race**					
Non-Hispanic white	1.00	**1.26 (1.09-1.47)**	**1.69 (1.41-2.02)**	**1.97 (1.53-2.55)**	0.023
Non-Hispanic black	1.00	1.06 (0.92-1.22)	1.17 (0.96-1.43)	**1.23 (1.02-1.50)**	
Hispanic	1.00	**1.46 (1.24-1.72)**	**1.80 (1.36-2.36)**	**1.75 (1.33-2.29)**	
Others	1.00	0.75 (0.42-1.34)	0.67 (0.40-1.11)	**1.91 (1.14-3.18)**	
**Ratio of family income to poverty**					
≤1.30	1.00	**1.29 (1.02-1.64)**	**1.37 (1.04-1.81)**	**1.44 (1.05-1.98)**	0.11
1.31-3.50	1.00	**1.19 (1.01-1.41)**	**1.57 (1.29-1.93)**	**2.08 (1.52-2.85)**	
>3.50	1.00	1.28 (0.98-1.68)	**1.78 (1.34-2.37)**	**1.78 (1.23-2.58)**	
**Smoking status**					
Non-smoker	1.00	1.15 (0.91-1.45)	**1.51 (1.21-1.87)**	**1.63 (1.26-2.10)**	0.0002
Former smoker	1.00	1.10 (0.92-1.31)	**1.39 (1.13-1.72)**	**1.96 (1.39-2.75)**	
Current smoking	1.00	**1.36 (1.11-1.68)**	**1.66 (1.31-2.11)**	**1.67 (1.27-2.19)**	
**Alcohol intake**					
Non-drinker	1.00	**1.21 (1.05-1.41)**	**1.51 (1.28-1.79)**	**1.87 (1.51-2.31)**	0.25
Current drinker	1.00	1.28 (0.996-1.64)	**1.74 (1.24-2.44)**	1.29 (0.99-1.67)	

Supplemental Table 1. Association Between Nocturia and All-Cause Mortality among Different Subgroups (continued)

	No. Nocturia Episodes				*P* interaction
	None	Once	Twice	Three or more times	
**Physical Activity**					
Inactive or insufficient	1.00	**1.25 (1.10-1.43)**	**1.60 (1.35-1.90)**	**1.91 (1.52-2.41)**	0.52
Recommended level	1.00	1.21 (0.98-1.50)	**1.45 (1.16-1.81)**	**1.67 (1.24-2.24)**	
**BMI categories (kg/m^2)**					
BMI < 25.0	1.00	**1.43 (1.16-1.76)**	**1.57 (1.28-1.94)**	**2.20 (1.62-2.98)**	0.0039
25.0≤ BMI <30.0	1.00	1.14 (0.99-1.31)	**1.47 (1.23-1.76)**	**1.53 (1.17-2.01)**	
BMI ≥ 30.0	1.00	1.13 (0.93-1.36)	**1.48 (1.14-1.93)**	**1.64 (1.18-2.27)**	
**Hypertension**					
No	1.00	**1.26 (1.03-1.55)**	**1.39 (1.13-1.70)**	**1.93 (1.43-2.60)**	0.038
Yes	1.00	1.15 (0.99-1.34)	**1.60 (1.38-1.86)**	**1.63 (1.36-1.96)**	
**Diabetes mellitus**					
No	1.00	**1.31 (1.02-1.67)**	**1.57 (1.33-1.85)**	**1.79 (1.42-2.25)**	0.11
Yes	1.00	**1.19 (1.02-1.38)**	**1.39 (1.07-1.81)**	**1.70 (1.24-2.33)**	
**Dyslipidemia**					
No	1.00	**1.24 (1.00-1.54)**	**1.59 (1.28-1.97)**	**1.52 (1.13-2.06)**	0.50
Yes	1.00	**1.21 (1.02-1.44)**	**1.43 (1.18-1.74)**	**2.14 (1.67-2.74)**	
**CRP**					
≤1 mg/dL	1.00	**1.24 (1.06-1.45)**	**1.55 (1.33-1.81)**	**1.86 (1.50-2.30)**	0.22
>1 mg/dL	1.00	1.10 (0.79-1.54)	1.26 (0.81,1.97)	**1.73 (1.15-2.60)**	

Hazard ratios were adjusted for age, sex, race/ethnicity, marital status, family income level, smoking status, alcohol intake, physical activity, total energy intake, overall diet quality indicated by HEI-2010, BMI, hypertension (yes/no), diabetes mellitus (yes/no), dyslipidemia (yes/no), and serum CRP.

Supplemental Table 2. Association Between Nocturia and Cardiovascular Mortality among Different Subgroups

	**No. Nocturia Episodes**				***P* interaction**
	**None**	**Once**	**Twice**	**Three or more times**	
**Sex**					
Men	1.00	**1.36 (1.04-1.79)**	**1.82 (1.30-2.54)**	**2.26 (1.44,3.54)**	0.13
Women	1.00	1.1 (0.84-1.45)	1.26 (0.90-1.76)	**1.77 (1.29-2.42)**	
**Age (years)**					
20-40	1.00	**2.52 (1.36-4.67)**	**2.10 (1.11-4.00)**	**3.25 (1.41-7.52)**	<.0001
40-60	1.00	1.28 (0.87-1.87)	1.54 (0.91-2.62)	**2.12 (1.28-3.52)**	
>60	1.00	1.00 (0.83-1.22)	1.28 (0.98-1.66)	**1.66 (1.28-2.15)**	
**Race**					
Non-Hispanic white	1.00	1.25 (0.97-1.60)	**1.61 (1.12-2.32)**	**2.23 (1.61-3.08)**	0.33
Non-Hispanic black	1.00	0.91 (0.68-1.23)	0.96 (0.68-1.35)	1.23 (0.89-1.70)	
Hispanic	1.00	**1.80 (1.28-2.53)**	**1.82 (1.15-2.89)**	**1.92 (1.18-3.12)**	
Others	1.00	1.24 (0.47-3.26)	0.85 (0.29-2.50)	2.51 (0.80-7.84)	
**Ratio of family income to poverty**					
≤1.30	1.00	1.50 (0.94-2.39)	**1.79 (1.11-2.87)**	**1.96 (1.24-3.09)**	0.78
1.31-3.50	1.00	**1.24 (1.00-1.54)**	**1.59 (1.11-2.28)**	**2.10 (1.42-3.10)**	
>3.50	1.00	0.90 (0.65-1.24)	1.16 (0.78-1.74)	1.70 (0.98-2.94)	
**Smoking status**					
Non-smoker	1.00	0.98 (0.75-1.27)	**1.41 (1.02-1.95)**	**1.77 (1.30-2.40)**	0.89
Former smoker	1.00	1.03 (0.80-1.32)	1.36 (0.87-2.12)	**1.89 (1.12-3.17)**	
Current smoking	1.00	**1.80 (1.11-2.92)**	1.59 (0.89-2.86)	**2.19 (1.36-3.51)**	
**Alcohol intake**					
Non-drinker	1.00	1.26 (0.995-1.59)	**1.44 (1.10-1.90)**	**2.15 (1.61-2.86)**	0.84
Current drinker	1.00	1.01 (0.60-1.70)	2.03 (0.997-4.12)	1.50 (0.70-3.18)	

Supplemental Table 2. Association Between Nocturia and Cardiovascular Mortality among Different Subgroups (continued)

	No. Nocturia Episodes				***P* interaction**
	**None**	**Once**	**Twice**	**Three or more times**	
**Physical Activity**					0.28
Inactive or insufficient	1.00	**1.34 (1.02-1.77)**	**1.60 (1.11-2.30)**	**2.18 (1.58-3.02)**	
Recommended level	1.00	1.09 (0.81-1.46)	1.18 (0.82-1.70)	**1.72 (1.19-2.49)**	
**BMI categories (kg/m^2)**					0.025
BMI < 25.0	1.00	**1.51 (1.05-2.18)**	**1.73 (1.14-2.62)**	**2.44 (1.67-3.57)**	
25.0≤ BMI <30.0	1.00	1.14 (0.81-1.59)	1.39 (0.98-1.99)	**1.73 (1.09-2.76)**	
BMI ≥ 30.0	1.00	1.06 (0.78-1.45)	1.24 (0.86-1.79)	**1.74 (1.13-2.69)**	
**Hypertension**					0.011
No	1.00	**1.60 (1.24-2.08)**	**1.83 (1.24-2.71)**	**1.97 (1.36,2.84)**	
Yes	1.00	0.99 (0.75-1.29)	1.18 (0.88-1.58)	**1.84 (1.34-2.55)**	
**Diabetes mellitus**					0.11
No	1.00	1.22 (0.96-1.56)	**1.65 (1.18-2.29)**	**2.10 (1.69-2.61)**	
Yes	1.00	1.17 (0.83-1.65)	1.12 (0.81-1.55)	1.60 (0.94-2.71)	
**Dyslipidemia**					0.37
No	1.00	1.15 (0.79-1.65)	1.47 (0.93-2.34)	**1.99 (1.26-3.16)**	
Yes	1.00	**1.26 (1.02-1.57)**	**1.42 (1.06-1.90)**	**2.02 (1.42-2.88)**	
**CRP**					0.48
≤1 mg/dL	1.00	**1.23 (1.00-1.52)**	**1.53 (1.19-1.97)**	**1.96 (1.47-2.61)**	
**>1** mg/dL	1.00	1.55 (0.84,2.89)	1.41 (0.72,2.75)	**2.57 (1.10,5.98)**	

Hazard ratios were adjusted for age, sex, race/ethnicity, marital status, family income level, smoking status, alcohol intake, physical activity, total energy intake, overall diet quality indicated by HEI-2010, BMI, hypertension (yes/no), diabetes mellitus (yes/no), dyslipidemia (yes/no), and serum CRP.

Supplementary Table 3. Sensitivity analyses of the association between Nocturia and mortality excluding those who developed died within two years of follow-up, missing covariates and BMI ≥ 40.0 kg/m^2.(n=10371)

	No. Nocturia Episodes			
**Outcomes**	**None, n=4570**	**Once, n=3582**	**Twice, n=1412**	**Three or more times, n=807**
**All-cause mortality**	1.00	**1.29 (1.12, 1.49)**	**1.64 (1.38, 1.95)**	**1.82 (1.43, 2.31)**
**CVD mortality**	1.00	**1.29 (1.04, 1.61)**	**1.62 (1.16, 2.27)**	**2.13 (1.50, 3.02)**
**IHD mortality**	1.00	**1.41 (1.12, 1.77)**	**1.77 (1.27, 2.45)**	**2.23 (1.58, 3.15)**
**Stroke mortality**	1.00	0.86 (0.56, 1.33)	1.10 (0.49, 2.43)	1.61 (0.75, 3.45)

Data were presented as hazard ratios (95% CIs) with adjustment of age, sex, race/ethnicity, marital status, family income level, smoking status, alcohol intake, physical activity, TEI, and overall diet quality indicated by HEI-2010, BMI, hypertension (yes/no), diabetes mellitus (yes/no), and dyslipidemia (yes/no), CRP.

Abbreviations: HEI, healthy eating index; TEI, total energy intake; BMI, body mass index; CVD, cardiovascular disease; IHD, ischemic heart disease.

Supplementary Table 4. Association between Nocturia and cause-specific mortality in competing-risk models

**No. Nocturia Episodes**	**CVD**		**IHD**		**Stroke**	
	**Deaths, n=1720,**	**HR (95% CI)**	**Deaths, n =1395,**	**HR (95% CI)**	**Deaths, n = 325,**	**HR (95% CI)**
**None**	459	1.00	366	1.00	93	1.00
**Once**	615	1.12 (0.992, 1.27)	505	**1.17 (1.02, 1.34)**	110	0.94 (0.71, 1.25)
**Twice**	345	**1.23 (1.06, 1.42)**	278	**1.27 (1.08, 1.50)**	67	1.06 (0.76, 1.47)
**Three or more times**	301	**1.66 (1.42, 1.93)**	246	**1.73 (1.46, 2.05)**	55	1.38 (0.97, 1.96)

HR = hazard ratio. Multivariable model was fully adjusted for the same covariates as Model 4 in the Table3.

Due to data limitations, sampling parameters cannot be used in competitive risk analysis.

Abbreviations: CVD, cardiovascular disease; IHD, ischemic heart disease.
